# Design and Waveform Assessment of a Flexible-Structure-Based Inertia-Drive Motor

**DOI:** 10.3390/mi10110771

**Published:** 2019-11-12

**Authors:** Junyang Wei, Sergej Fatikow, Hai Li, Xianmin Zhang

**Affiliations:** 1 Division Microrobotics and Control Engineering, University of Oldenburg, 26129 Oldenburg, Germany; sergej.fatikow@uni-oldenburg.de; 2 Guangdong Provincial Key Laboratory of Precision Equipment and Manufacture Technology, South China University of Technology, Guangzhou 510640, China; lihai@scut.edu.cn (H.L.); zhangxm@scut.edu.cn (X.Z.)

**Keywords:** inertia drive, stick-slip motor, low-harmonic waveform, flexible structure

## Abstract

This paper reports the mechanical design, waveform investigation and experimental validation of an flexible-structure-based inertia-drive linear motor. The flexible structure is designed and verified with finite element analysis to meet the bandwidth requirement for high-frequency actuation. In order to improve the output velocity, non-resonance low-harmonic driving waveform is implemented and evaluated. Experimental results show that the motor is capable of an output velocity of 2.41 mm/s with the waveform, compared to 0.73 mm/s with the classic saw-tooth waveform actuation. The improvement of the non-resonance low-harmonic waveform for the flexible-structure-based motor is confirmed.

## 1. Introduction

Currently, the rapidly growing demand for micro/nano-manipulation is becoming more and more important to optical engineering, biomedical, micro/ nano-manufacturing and other areas. In order to meet the requirements including but not limited to miniaturization, high velocity, high precision and long travel range for micro/nano-manipulation tasks, numerous micro-motion piezoelectric motors have been developed and can be found extensively in different applications. The driving principles of piezoelectric motors can be classified into direct-drive, displacement-amplified-drive, ultrasonic-drive, inchworm-drive, inertia-drive, etc. Inertia-drive motors, also known as “stick-slip” motors, have the prominent merits of compact size and simple driving signal, attract many research attentions [1,2,3].

Pohl developed one of the first inertia-drive motors in 1987 [4]. Focusing on improving the output performance (mainly the output velocity) of the motors, waveform optimizations have been studied intensively. Parabolic, cycloid and exponential waveforms in addition to the classic saw-tooth waveform (STW) have been investigated and developed. Bergander proposed the implement of the input shaping method to reduce the residual vibration [5]. Nguyen presented an optimized waveform based on the method of dimensionality reduction to reduce the backlash [6]. Hunstig theoretically investigated the performance of frequency-limited waveforms [7]. Cheng proposed a resonant/off-resonant hybrid excitation waveform [8]. Zhong presented an combined waveform to increase the velocity [9].

From the perspective of structural optimization, simple forms of piezoelectric actuators such as bulk, stack, tube, bimorph structure were generally used for inertia-drive motors in the early stage development. Soon after, the concept of monolithic piezoelectric flexible structures, and the use of extra flexible structures were investigated, where the steps are generated through the deformation of the structures [10,11,12]. Motor designs that introduce flexible structures have the advantages of design flexibility, capability of easy replacement and integration of sensors, protection to the piezoelectric actuators, etc. However, flexible structures with relatively low stiffness were concluded as complex designs, which have low driving frequencies and lead to poor output velocities. Since then, researchers aimed at compact and simple designs in order to push the driving frequency limit. For example, the so-called “push-pull” actuator and similar concepts were proposed [13,14,15], which enables high frequency actuation for motors or microrobots. Their common characteristic is the small stroke or step, usually less than few hundreds nanometers. Until recent years, inertia-drive motors based on flexible structure designs have started to be proposed again [16,17,18]. Yet the focus is mostly on the novelty of structure design or driving principle. The issue of the limitation on the driving frequency and the output performance using flexible structures is not widely in discussion.

In a recent study [7], theoretical analysis has shown that except for a high driving frequency, a large stroke is also a key factor to achieve a high output velocity of an inertia-drive motor. That is to say, with the advantage of a large stroke, flexible-structure-based inertia-drive motors could possibly have high output velocities, if they have acceptable driving frequencies. The compromise between the stroke and the driving frequency plays a key role. Results from another field have proved the feasibility of utilizing flexible structures in high frequency actuations [19,20].

To achieve a high driving frequency, a narrow frequency-spectrum driving signal is as well a possible solution. For inertia-drive, a related reported signal is the low-harmonic waveform (LHW). It is composed of only two harmonic components, the second of which has twice the frequency of the first. Compared to the STW, the LHW has a narrow frequency-spectrum. Nevertheless, it is mostly implemented and studied in resonance actuation at ultrahigh-frequencies (usually higher than 10 kHz) [21,22,23,24]. Such driving frequency is too high for general flexible structure design and the actuation intrinsic is different. A non-resonance actuation with a maximum driving frequency below few thousand Hz is more reasonable for flexible-structure-based inertia-drive motor. However, the study of non-resonance LHW actuation, especially its implementation on flexible structures is few. Its performance in this frequency region remains to be investigated.

In the scope of this paper, the main contribution is to verify the feasibility of the non-resonance LHW actuation in an inertia-drive motor that based on a high-bandwidth flexible structure. The performance of the non-resonance LHW actuation will be validated in compare with the STW actuation, investigating its potential to improve the driving frequency and output velocity of the motor.

## 2. Driving Principle

**Figure 1:**
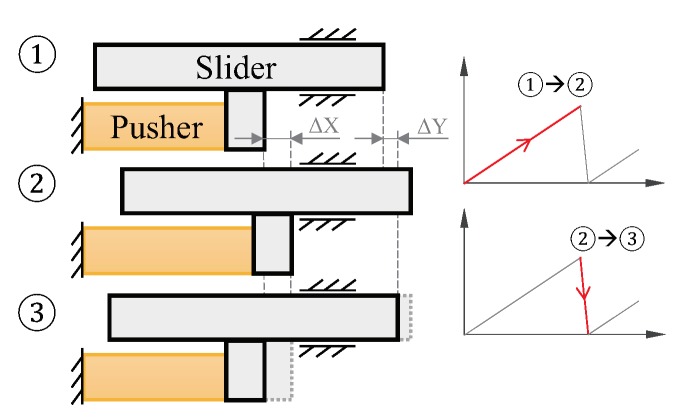
Driving principle of a linear inertia-drive motor. The transition from ① to ② represents the stick phase, while ② to ③ represents the slip phase. The curve graph on the right represents the displacement of the pusher part vs. time.

An inertia-drive motor in general is composed of two parts, the pusher part that generates displacement corresponding to the driving signal, and the slider part that represents the final output displacement of the complete motor. Figure 1 illustrates the driving principle of such a linear motor. Applying the STW signal as the driving signal, a net output step (ΔY) can be generated in one period. This process contains two phases, the stick phase and the slip phase. In the stick phase, the pusher expands slowly, generating the driving step (ΔX). The slider moves together with the pusher due to the static friction force. Followed by the slip phase, the pusher then contracts rapidly to its original position, while the slider fails to stick together because the maximum static friction force does not exceed the inertia force. Consequently the net output step is created. By executing this period continuously, the macro output displacement of the motor is generated.

However, such a driving signal has a shortcoming. The residual vibration appearing after each slip phase hinders the output performance of the inertia-drive motor, especially when driven with high frequency signals. As known, an arbitrary periodic signal can be described as a Fourier series with infinite harmonic terms. Specifically, for a STW with an amplitude A and period T, its function during one period (start at the origin) can be represented as

(1) $$\begin{array}{c} f\left(t\right)=\frac{At}{T},\;0\leq t\leq T \end{array}$$

The Fourier series of Equation (1) on the interval [0,T] is therefore given by

(2) $$\begin{array}{c} f\left(t\right)=\frac{A}{2}-\frac{A}{\pi }\sum_{n=1}^{\infty }\frac{\sin(n\omega t)}{n},\;n=1,2,3\ldots \end{array}$$

It is expected that some of the high frequency components mechanically excite the resonances of the pusher part and creates residual vibrations. Thus, it is advantageous to adopt fewer frequency components only. Taking the first two harmonics in Equation (2) and rewriting the equation, the 2-term LHW is

(3) $$\begin{array}{c} f_{LHW}\left(t\right)=-\frac{A_{L}}{\pi }\left[\sin\left(\omega t\right)+\frac{1}{R_{l}}\sin\left(2\omega t-\theta \right)\right] \end{array}$$

where $A_{L}$ is the amplitude for the LHW, $R_{l}$ is the ratio coefficient of the second harmonic, and θ is the phase shift of the second harmonic. A 2-term low-harmonic LHW is theoretically capable of driving the pusher up to half its first resonance frequency before exciting the resonance. The best parameters for driving inertia-drive motor are 4 to 1 of the amplitude ratio for the two harmonics, and no phase shift of the second harmonic [21]. Thus in this paper, a LHW with a ratio coefficient of 4 and a phase shift of 0 is in focus.

It should be noted that, driven with LHWs, the intrinsic nature of the driving becomes different, which no longer contains only the two “stick” and ”slip” phases. Few of research has cover this issue so far, one can be seen in [7].

## 3. Mechanical Design

### 3.1. Design Consideration

**Figure 2:**
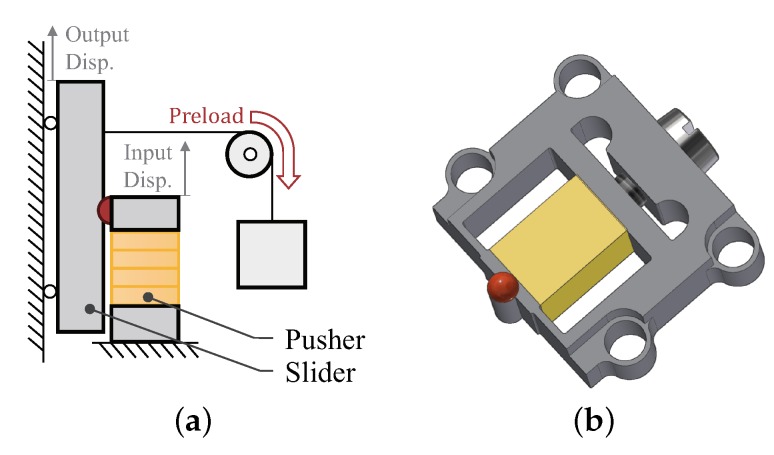
Schematic illustration of the motor setup. (**a**) Mechanical structure illustration of the motor; (**b**) Compositions of the pusher part.

As shown in Figure 2a, the motor setup is composed of a pusher part, a slider part and an extra preload adjustment mechanism. For the pusher part, a simple flexible structure has been designed, as illustrated in Figure 2b. Piezoelectric stacks are widely used to drive flexible structures, as they not only provide large driving force, but also generate precise displacements with a high acceleration over a high mechanical bandwidth. Thus, a commercial piezoelectric stack (Piezomechanik GmbH, Pst-150, München, Germany) is selected, whose technique parameters can be found in Table 1. The stack is nested in the slot and pressed against a small block at one end. A screw providing pre-tightening force at the other end of the piezoelectric stack. With a contact tip attached to the top face, the block functions as the driving end of the pusher, i.e., generates the input displacement. A pair of leaf flexible hinges are applied between the block and the support frame which provides protection and guidance to the piezoelectric stack. Please note that the flexible structure as a study case in this paper, can be designed with other types of hinges or replaced by more complex structure in actual applications.

To achieve a high driving frequency, the flexible structure needs to be stiffness enough to provide a high mechanical bandwidth, meanwhile not hindering too much the effective stroke of the piezoelectric stack. The parameters of the leaf flexible hinges affect greatly the dynamic behavior of the flexible structure, i.e., the pusher and subsequently the output performance of the motor. As the thickness and length been fixed for the purpose of compact design, the width of the hinges is selected as the variable for the first resonance frequency determination.

Aiming at a possible maximum driving frequency in an interval between 1 kHz and 3 kHz, the first resonance frequency of the flexible structure should be at minimum ten times larger in value [19]. Based on the knowledge of mechanics of materials, if the pair of hinges is simplified as a fixed-fixed beam with distributed mass, and let its first resonance frequency *f* in an interval between 10 kHz and 30 kHz, a theoretical estimation of the width for the leaf flexible hinges is calculated using the equation given by [25]

(4) $$\begin{array}{c} f=\frac{K_{1}}{2\pi }\sqrt{\frac{\mathrm{EI}}{\rho {\mathrm{SL}}^{4}}} \end{array}$$

where $K_{1}=22.4$ is a constant for the first resonance frequency, ρ is the density of the beam material, S is the sectional area of the beam, L is the length of the beam and EI is the flexural rigidity of the beam. The interval of the width of the hinges, i.e., the height of the 12 mm long beam with modulus of elasticity 72 Gpa and density 2.7 $g/{\mathrm{cm}}^{3}$ can be calculated as from 0.28 mm to 0.83 mm. Any width value in this interval satisfies the bandwidth requirement. We choose the value 0.5 mm for the hinges width, with which the first resonance frequency is 18091 Hz by calculation.

Next, to check if the stiffness of the hinges hinders the effective stroke of the piezoelectric stack, it is calculated to compare with the stiffness of the latter. In this case, the value is $K_{h}=\left(2\times 48\mathrm{EI}\right)/L^{3}\approx 15.8$ N/μm, which is an acceptable result that is much less than the value of the piezoelectric stack, 120 N/μm.

### 3.2. Finite Element Analysis

**Figure 3:**
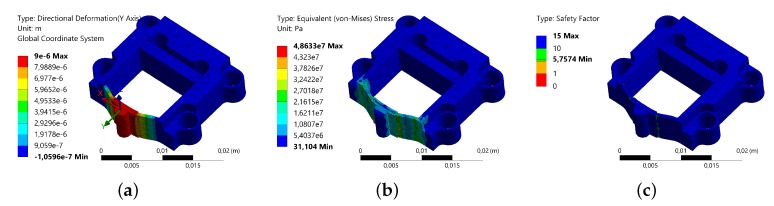
Static analysis results of the flexible structure. (**a**) Deformation of the hinges in the actuating direction; (**b**) Distribution of the equivalent stress; (**c**) Distribution of the safety factor.

**Figure 4:**
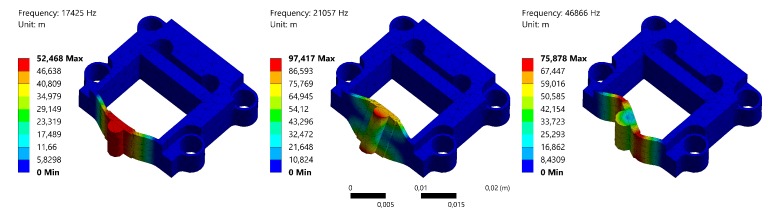
The first three dynamic modals of the flexible structure.

For the purpose of confirming the design validity, the finite element analysis (FEA) simulation is conducted. The material utilized in the simulation is Aluminum Alloy, whose parameters are set to be the same as above. Finite element Solid 187 is used. The mounting holes are set as fixed boundary faces. The static analysis shows the deformation of the flexible structure in the same direction of the piezoelectric stack elongation. Undesired motions in other direction are not observed. The equivalent stress and safety factor are checked to avoid material failure of the structure. As shown in Figure 3, the threshold deformation of 9 μm (stroke of the piezoelectric stack) for the flexible structure is investigated. The results indicate that the maximum equivalent stress is 48.6 Mpa, which is smaller than the allowable tensile stress (0.28 Gpa) of the material. A minimum safety factor of 5.8 is obtained. In order to ensure a high bandwidth, the modal analysis is carried out to obtain the dynamic behaviors of the flexible structure, which reveal its first three modals. The results are presented in Figure 4, as the resonance frequency values are 17,425 Hz, 21,057 Hz and 46,866 Hz separately, indicating a high-bandwidth design of the flexible structure. It can be seen that the first modal is in-plane vibration, whose frequency is close to the value calculated by Equation (4). Yet the value is slightly smaller, as the equation neglects the center mass, i.e., the block mass.

## 4. Experiments and Discussion

### 4.1. Identification and Modelling of the Pusher

For the pusher part, the first resonance frequency is our main concern. A model of the pusher in the mechanical aspect helps to understand and further to compensate the driving displacement, especially in the high frequency region approaching the first resonance frequency. Based on previous research, the dynamic characteristic of the piezoelectric stack is equivalent to a mass-spring-damping system, if the hysteresis and creep are neglected. The same also applied to the flexible structure under small deformation assumption. Thus, the pusher can be represented as a system that the two connected in series and further simplified as a general mass-spring-damping system.

**Figure 5:**
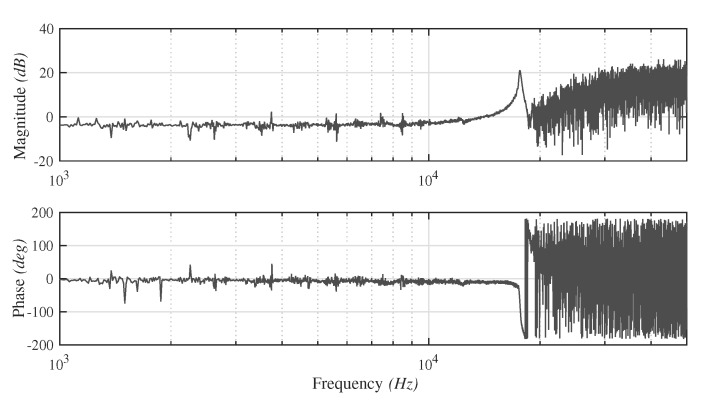
Frequency response of the pusher in the experimental setup.

A frequency response measurement of the experimental setup (see Section 4.2) was carried out to characterize the dynamics of the pusher part. Due to the sample rate limitation of the measurement equipment, data with a driving frequency above 50 kHz cannot be examined. Figure 5 shows the Bode plot of the experimental data, which shows the first resonance frequency at 17,646.3 Hz and confirms the validity of the simulation results.

Given the assumed condition, a transfer function model G(s) is estimated for the pusher using system identification toolbox in Matlab, as

(5) $$\begin{array}{c} G\left(s\right)=\frac{7.5\times 10^{9}}{s^{2}+2000s+1.24\times 10^{10}} \end{array}$$

### 4.2. Experimental Setup

**Figure 6:**
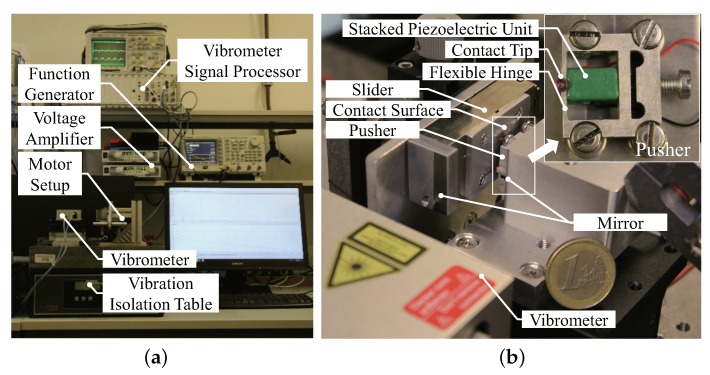
Experimental setup including the motor prototype. (**a**) Overall view of the experimental setup system; (**b**) Detail view of the motor prototype including the pusher.

**Figure 7:**
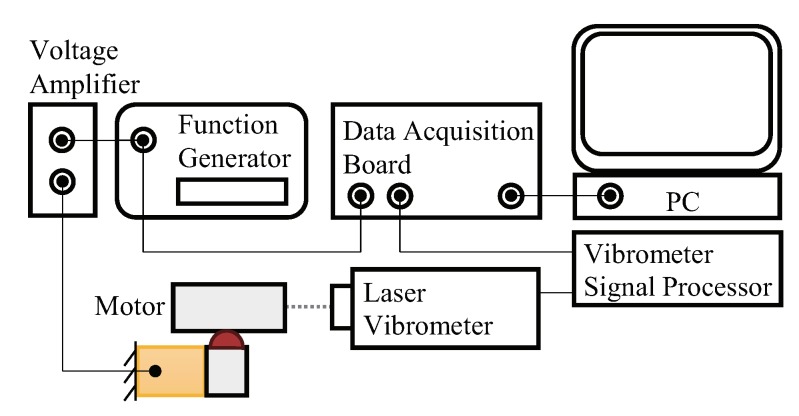
Schematic control graph of the experimental setup.

As shown in Figure 6a, the experimental setup is consisted of the motor prototype, a function generator (AFG3102, Tektronix, Beaverton, OR, USA), a customized voltage amplifier with an amplification ratio of 18.65, a vibration isolation table (Micro40, Halcyonics GmbH, Göttingen, Germany), a vibrometer system (SP120, SIOS Metechnik GmbH, Ilmenau, Germany) and a PC with a data acquisition board (PCI-6229, NI, Austin, TX, USA) which is not shown. The schematic control graph of the experimental setup is shown in Figure 7.

Presented in Figure 6b, the motor prototype includes the driving component, i.e., the pusher and the motion component, i.e., the slider. They are fixed on the vibration isolation table separately via two positioning stage. The flexible structure is manufactured with a wire electrical discharge machine (A280, Sodick GmbH, Düsseldorf, Germany) using aluminum alloy material. The slider is constrained by a linear bearing and an aluminum alloy plate attached on top functions as the contact surface. An extra linear bearing provides single degree-of-freedom movement normal to the contacting surface, so that a pulley and a weight block can be utilized to provide adjustable preload against the contact tip of the driving component. Unless specifically mentioned, the preload used below is 1.2 N.

### 4.3. Results and Discussion

#### 4.3.1. Input Displacement

**Figure 8:**
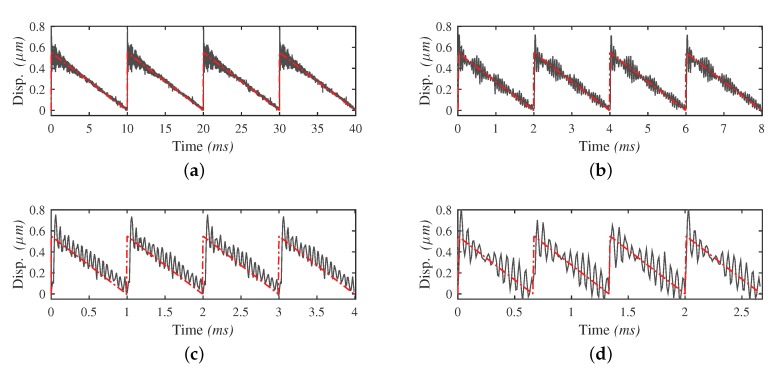
Input displacements with STW signals in different driving frequencies. The grey curves represent the actual input displacement of the pusher, and the red curves represent the ideal input displacement driven by STW signals. Due to the residual vibration, the displacement is flipped for better visualization. The frequencies of the signals are: (**a**) 100 Hz; (**b**) 500 Hz; (**c**) 1000 Hz; (**d**) 1500 Hz.

Before measuring the output performance of the motor prototype, the characteristics of the input displacement is investigated. For the measurement of the input displacement, a mirror is mounted perpendicular to the displacement direction at a side of the block. The vibrometer then measures the data with laser reflected from the mirror. The sample rate in the measurement is 20 kHz. Starting with a voltage amplitude of 0.5 V, two waveforms of driving signals in a wide range of frequency bandwidth from 0 Hz to half the first resonance frequency of the structure are tested. Figure 8 demonstrates the input displacements utilizing the STW signals at different frequencies. As shown, residual vibration can be obviously observed. The proportion of the residual vibration damp-out time to the stick phase duration increases as the driving frequency rises. When the frequency value is beyond 400 Hz, the time needed to damp the residual vibration exceeds the duration of the stick phase. With higher frequency, the vibration further distorts the displacement and undermines the stick condition of the contact tip and surface in the stick phase, leading to a weakened output performance.

**Figure 9:**
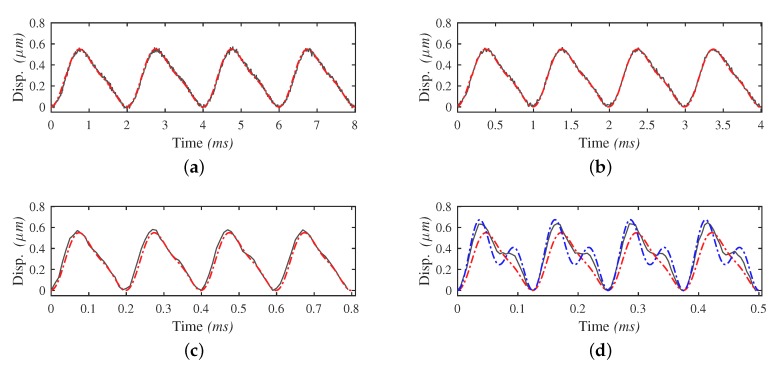
Input displacements with the LHW signals in different driving frequencies. The grey curves represent the actual input displacements of the pusher part, and the red curves represent the ideal input displacements driven by STW signals. The frequencies of the signals are: (**a**) 500 Hz; (**b**) 1000 Hz; (**c**) 5000 Hz; (**d**) 8000 Hz.

As a comparison, Figure 9 demonstrates the input displacement with the LHW signals applied in a wide range of frequency bandwidth from 0 Hz to half the first resonance frequency. Thanks to the sharp frequency spectrum, input displacement at a driving frequency of more than 8000 Hz is achieved, which is closed to half the first resonance frequency of the pusher part. No unpredicted displacement distortion nor residual vibration is observed except the noise induced by the measurement equipment and the environment. The actual input displacements are in good accordance with the ideal input displacements in a wide frequency range.

It should be noted that, in the frequency range closed to half the first resonance frequency, i.e., when the frequency of the second harmonic component is close to excite the resonance, the amplitude of the second-term displacement of the input displacement corresponded to the second harmonic component of the waveform is rapidly magnified to far beyond the first-term displacement. Thus the actual amplitude ratio of the two harmonic terms in the input displacement is changed and causes waveform distortion, as shown in Figure 9d. This distortion can be predicted utilizing the model of the pusher. The curve in blue is the simulated input displacement calculated with the model in Section 4.1. It shows better prediction of the displacement amplitude and indicates the distortion tendency. However, the curve does not fit perfectly. The reason is that the second-order model identified in this case cannot fully capture the dynamics of the pusher. The frequency response magnitude of the model in the near-nature-frequency range is higher than that in the actual experimental setup, thus leads to a result with smaller ratio coefficient.

**Figure 10:**
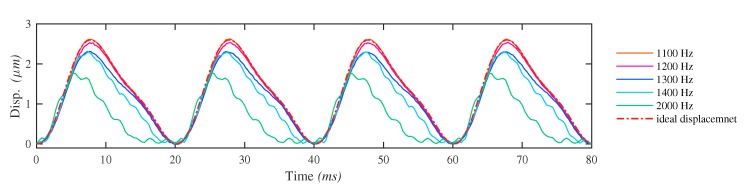
Distortion of the input displacements with the LHW signals at different driving frequencies. The voltage amplitude is 2.3 V. All the time vectors of the displacements at different frequencies have been remapped to a same frequency (f* = 50 Hz) for better distortion comparison.

In the following step, the voltage amplitude of the signal is increased to check the consistency. For STWs, the results show great consistency. The residual vibrations impact greatly the shapes of the input displacements, leading to worse distortions as the frequency increase. On the other hand, for LHWs, when a certain frequency threshold is reached, unexpected distortions of the input displacement along with abrupt grown sound noise are observed. The distortions happen at the bottom of the curves, presenting a saturation feature. As the voltage rises in amplitude, the frequency threshold for undistorted displacements drops. For example, with a voltage amplitude of 1.5 V, the frequency threshold for distortion is around 2100–2300 Hz. With a voltage amplitude of 2.3 V, it is around 1200–1400 Hz. To be more specific, at around 1200 Hz the displacement amplitude starts to decrease. At around 1300 Hz the distortion appears occasionally and at above 1400 Hz the distortion happens regularly and becomes greater as the frequency increases. Input displacements with frequency higher than the threshold are not implemented in the later driving tests, since they are distorted and cannot represent correctly the output performance of LHWs. Figure 10 illustrates the distortions of the input displacements at frequencies near the frequency threshold.

#### 4.3.2. Output Displacement

The output performance of the motor prototype is tested using the two types of waveforms. Voltage amplitudes of 0.5 V and 2.3 V are investigated. For the voltage amplitude of 0.5 V, the minimum output displacement can be observed clearly using STW signals at the frequency less than 1100 Hz. Higher frequencies cannot produce output displacements but vibrations. Meanwhile using LHW signals, no obvious output displacement is obtained, whatsoever the driving frequency is. The measured data show only vibrations whose frequencies are mostly the same as the ones of the driving signals.

**Figure 11:**
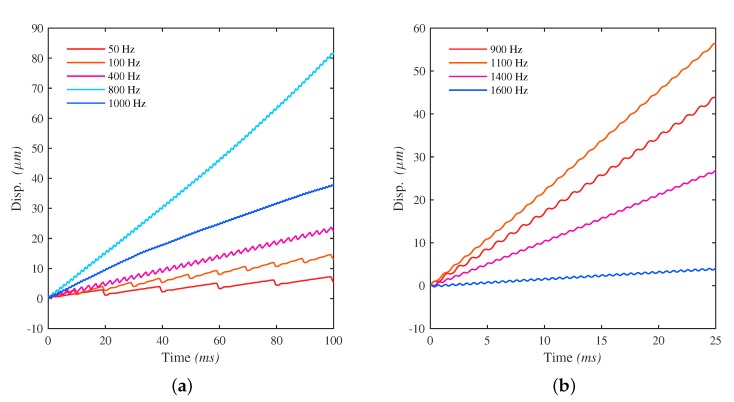
The output displacements at different frequencies driven by waveform: (**a**) STW; (**b**) LHW.

Next, the voltage amplitude of 2.3 V is tested. Output displacements can be observed using both types of waveforms. Figure 11 shows the relationship between the output displacement and the time at different frequencies for the two types of waveforms. For the output displacements using STW signals, the typical two-phase movement “stick and slip” can be identified clearly at frequencies below 100 Hz. The residual vibration has minor effect on the output displacement. As frequency goes higher, the effect of the residual vibration appears: the net output step becomes smaller, the two-phase movement can no longer be recognized and the out displacement curve becomes more “smooth” and more unstable. In comparison with STW, output displacements using LHW signals do not contains the typical stick and slip phases. At their workable frequency range, the curves always appear as smooth stair-like movements, but their net output steps become smaller and smaller beyond 1100 Hz.

**Figure 12:**
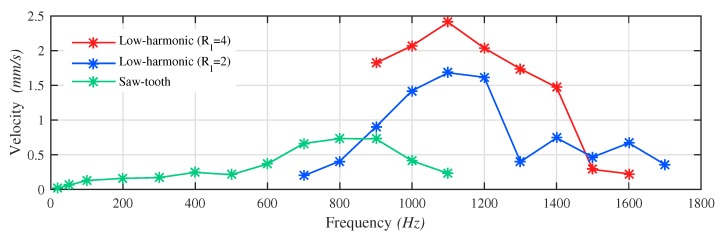
The average output velocity vs. the driving frequency in two types of waveforms. Each value of every average output velocity point is calculated from 3–5 sets of displacement data.

Figure 12 depicts the overall relationship between the average output velocity and the driving frequency of the two types of waveforms. For the STW, the figure indicates that the average output velocity grows as the frequency increases. However, the linearity is not ideal due to the disturbances of the residual vibration, as it hinders the “stick phase” and causes unstable output steps and consequently the output velocity. The peak value of 0.73 mm/s is reached at 800 Hz. Beyond this frequency, the effect of the residual vibration gradually overwhelms the effect of the driving signal and leads to a drop of the velocity. Beyond 1100 Hz, the velocity declines to 0 mm/s which can be regard as total failures of the STW due to distortions. For the LHW, the workable frequency range starts at 900 Hz. Below this frequency no output velocity is observed. The average output velocity increases linearly with the frequency until 1100 Hz, where the maximum value of the curve is 2.41 mm/s. After that, the curve decreases. The results in the authors’ opinion, is relevant to the unexpected distortion of the input displacement investigated in Section 4.3.1. The distortion appears at 1200 Hz, which is in agreement with the frequency when the curve starts to decline. It changes the shape of the waveform and thus the driving principle, undermining the driving efficiency. Without the distortion, a further increment of the velocity curve is expected according to the theoretical analysis. Additionally, another LHW signal with a ratio coefficient $R_{l}=2$ and a phase shift θ=0 is also implemented in the experiments for comparison. The results show similar trends and also prove the conclusion of former research that its performance is inferior to the one in the experiment before. Overall, the LHW has better performance than the STW with a voltage amplitude of 2.3 V.

#### 4.3.3. Discussion

As shown in the former subsections, the results demonstrate that the LHW is superior to the STW under certain conditions and it has great potentials to achieve better performance. According to the experimental results, two questions need to be discussed.

- Why the setup does not have any output displacement utilizing 0.5 V LHW driving signals? The same question goes for the 2.3 V signals at frequencies lower than 900 Hz.
- Is it possible that the decline of the velocity, which represents the performance of LHWs in Figure 12, comes from residual vibrations excited by the waveform signals (rather than the unexpected distortion that is but one special example in this case)? Therefore, it may prove the mistake of the theory? If not, where does the distortion of the input displacement come from?

For the first question, the reason likely comes from the intrinsic nature of the driving principle. As mentioned earlier, a motor driven by LHW signals may no longer contains the typical ‘stick’ and ‘slip’ phases. The complex phenomenon involves many different factors including the frequency, the voltage, the preload force, etc., and it has not yet been clearly investigated. According to the analysis in [7], it is summarized as “slip-slip actuation”. Below certain frequencies or voltages, the acceleration provided may not meet the requirement to achieve the duo-slipping condition to produce a net step. Input displacements utilizing LHW signals in most related research have amplitudes of more than several micrometers, i.e., in micro-scale, especially those who take advantage of resonance vibrations. With a 0.5 V waveform signal, the amplitude of the input displacement is in nano-scale, which may not have an acceleration large enough to overcome the maximum static force to generate a slip. In addition, the contact surface in nano-scale could lead to interesting and sophisticated friction phenomena. Pre-sliding zone may appear due to the elastic deformation of the surface, or the surface asperities could cause deviations of the contact. Besides, the mechanical structure of the experimental setup is composed of many parts including the bearing, which inevitably has clearances. Other nonlinear factors such as the creep and hysteresis of the piezoelectric material may as well play a role. Therefore, it is hard to draw a conclusion what the specific reason is. Future research is required.

For the second question, the experimental result of the 0.5 V LHWs has shown the capability of the frequency limitation this waveform can achieve. The result demonstrates well the theoretical prediction and no residual vibration occurs. A LHW in general contains the first two harmonics of a STW. This fact determines that it only produces resonance vibration (if counted as residual vibration) when the frequency of its second harmonics is high enough to excite the first resonance of the system, while the nth harmonics of the STW inevitably excite multiple resonances. This shows the superiority of utilizing the LHW over the STW at the aspect of high frequency actuation. The reason why the unexpected distortion happens, probably comes from the mounting method of the piezoelectric stack. It is clamped tightly through pre-tightening force. With a relatively high voltage, the acceleration during the elongation or contraction process of the piezoelectric stack may exceed the maximum force that the pre-tightening force can provide, thus produces a possible gap and impacts the results consequently. A more reliable mounting method will be implemented in the future.

Overall, the results show that compared to the STW, the LHW can improve the output performance when the input displacement is reasonably large. It eliminates the residual vibration that excited in the STW and therefore covers higher driving frequency ranges. This is meaningful for actuators designed with flexible structures as it broadens the scope of applications. In fact, similar effects can also be achieved utilizing low-pass filters from the perspective of control theory. Yet, the LHW provides a more direct and easy-access method and promotes the area of driving principle for inertia-drive motors or usage in microrobotics. In the future, the design of the setup system will be improved, so that a more systematic and coherent investigation for LHW can be presented.

## 5. Conclusions

In this paper, the mechanical design, driving waveform investigation, experimental validation and discussion of a piezoelectric inertia-drive motor have been presented. Through analytical calculation and FEA, a decent dynamic performance of the pusher is ensured. A mass-spring-damping system model has been established, meant to predict the input displacement of the pusher part in the near region of the first resonance frequency. In order to improve the output velocity, the non-resonance low-harmonic driving waveform is adopted and compared with the classic saw-tooth driving waveform. Experimental results show that with the STW, the motor is capable of achieving a maximum output velocity of 0.73 mm/s at 800 Hz and a maximum driving frequency with valid output of 1100 Hz. While utilizing the LHW the values are 2.41 mm/s at 1100 Hz and 1700 Hz, respectively. It is discussed and confirmed that the non-resonance LHW can improve the output performance and broaden the workable frequency range of the flexible-structure-based inertia-drive motor. The results show more application possibilities for flexible-structure-based designs working at dynamic conditions.

In future work, the overall experimental setup system especially the mechanical structure will be improved to solve the issues including the unexpected distortions of the input displacements when using high voltage LHW signals at high frequencies, and likely the unobserved output displacement issue using low voltage LHW signals. A deeper and more systematic investigation for the intrinsic nature of the driving utilizing LHW signals, is required to explain the unclarified phenomenon in the experiments. With a more in-depth study the potential of this type of waveform can be exploited further.

## Figures and Tables

**Table 1:** Data sheet of the piezoelectric stack used in the motor.

Dimension (mm)	Stroke (μm)	Stiffness (N/μm)	Resonance Frequency (kHz)
5×5×7	9	120	100

## Abbreviations

The following abbreviations are used in this manuscript:

STW	Saw-Tooth Waveform
LHW	Low-Harmonic Waveform
FEA	Finite Element Analysis
